# Spatiotemporal Evolution and Drivers of Highway Surface Deformation Based on SBAS-InSAR and Geodetector

**DOI:** 10.3390/s26113548

**Published:** 2026-06-03

**Authors:** Zhaoyang Chen, Jin Li, Xu Zhang, Junwei Bi

**Affiliations:** 1 Shandong Key Laboratory of Technologies and Systems for Intelligent Construction Equipment, Shandong Jiaotong University, Jinan 250357, China; 2 School of Qilu Transportation, Shandong University, Jinan 250002, China; 3 Department of Geotechnical Engineering, College of Civil Engineering, Tongji University, Shanghai 200092, China

**Keywords:** SBAS-InSAR, Geodetector, surface deformation, highway

## Abstract

**Highlights:**

- SBAS-InSAR quantifies highway deformation, revealing settlement rates up to −45 mm/a and five representative deformation zones.
- Fault distance and soil moisture show higher explanatory power, while multi-factor interactions further enhance deformation heterogeneity.
- The integrated approach supports targeted hazard assessment for infrastructure in plateau frozen-ground regions.

**Abstract:**

To address the lack of long-term, wide-area surface deformation observations along the geologically complex Dangxiong–Yangbajing section of the G6 Expressway in the frozen-ground region of the Qinghai–Tibet Plateau, where conventional monitoring is insufficient, we applied Small Baseline Subset Interferometric Synthetic Aperture Radar (SBAS-InSAR) to retrieve surface deformation within a 2.0 km corridor on both sides of the highway from 24 November 2021 to 26 December 2024, and to characterize the spatiotemporal evolution of deformation. We then integrated eight explanatory factors (slope, surface roughness, distance to rivers, distance to faults, surface soil moisture, precipitation, land surface temperature (LST), and fractional vegetation cover (FVC)). Geodetector was used to quantify their explanatory power and spatial heterogeneity with respect to deformation. The results show pronounced spatially uneven settlement along this highway segment, with maximum annual settlement rates exceeding −45 mm/a. Five settlement centers were identified, including two major pavement subsidence zones. Distance to faults and soil moisture showed higher single-factor explanatory power, whereas FVC, precipitation, and LST also contributed to deformation heterogeneity. Interaction detection further indicated that the interactions between fault-related conditions with vegetation, soil moisture, precipitation, and LST substantially enhanced the explanatory power, suggesting that the deformation pattern was associated with multi-factor coupling rather than a single dominant environmental factor. These findings demonstrate the utility of integrating SBAS-InSAR with Geodetector analysis for corridor-scale highway deformation assessment and provide a remote sensing basis for targeted hazard assessment and risk mitigation for highways in frozen-ground environments.

## 1. Introduction

Freeze–thaw processes are widespread in high-altitude and high-latitude frozen-ground environments [1]. For linear infrastructure traversing such regions, road subgrades and slopes are repeatedly affected by coupled thermal and hydrological processes, which can lead to frost heave, thaw settlement, fissuring, and differential settlement [2]. Accordingly, surface deformation exhibits characteristics of concealment, gradual progression, and pronounced spatial variation, readily accumulating damage during specific periods or at localized sections [3]. This compromises highway structural stability and traffic safety while escalating operational maintenance costs. In this context, large-scale and high-precision surface subsidence monitoring and analysis can provide important support for the prevention and control of geological disasters.

Traditional surface deformation monitoring commonly employs leveling surveys, Global Navigation Satellite System (GNSS), settlement plates, monitoring stakes, ground-penetrating radar, and field inspections [4]. Although these methods provide high accuracy at discrete locations, they often cannot support long-term, wide-area, continuous deformation monitoring in high-altitude cold regions. These limitations arise from constraints related to traffic access, sparse instrument deployment, high maintenance costs, and harsh meteorological conditions. Consequently, there is a pressing need for a non-contact monitoring approach that can provide continuous, large-scale deformation measurements with minimal field dependence.

To meet this need, satellite-based Interferometric Synthetic Aperture Radar (InSAR) has emerged as a powerful alternative to traditional ground surveys [5]. Early spaceborne Synthetic Aperture Radar (SAR) missions such as Seasat produced foundational datasets that enabled the subsequent development of interferometric applications. Regular satellite revisit cycles enable wide-area, efficient, and continuous Earth observation [6]. With its all-weather, day-and-night imaging capability, broad coverage, and high-precision measurements, InSAR has become a key tool for geological hazard monitoring and early warning worldwide [7,8]. In the InSAR methodology, Differential Interferometric Synthetic Aperture Radar (D-InSAR) technique [9] has been widely used to detect and monitor ground-surface movements associated with natural geologic processes (e.g., earthquakes [10] and landslides [11] as well as anthropogenic activities such as underground mining [12] and groundwater extraction [13]. D-InSAR offers clear advantages over conventional geodetic approaches, including wide spatial coverage, high imaging resolution, and non-contact measurements. However, its effectiveness for deformation monitoring of specialized narrow-strip infrastructures (e.g., highways) is often limited by spatio-temporal decorrelation and atmospheric delay effects [14]. Compared with conventional D-InSAR, time-series InSAR leverages multiple acquisitions to suppress atmospheric artifacts and alleviate spatiotemporal decorrelation, thereby improving deformation estimation accuracy to the millimeter level. In recent years, the field has shown rapid development and increasing maturity, with a growing body of work focusing on InSAR applications in transportation [15]. Among the most commonly used time-series InSAR techniques, SBAS-InSAR [16] and Permanent Scatterer InSAR (PS-InSAR) [17] have been extensively investigated for retrieving deformation time series over man-made infrastructures such as railways, highways, and bridges [18,19,20].

Beyond deformation retrieval, recent research increasingly couples time-series InSAR with hazard identification and corridor-scale risk-oriented assessment, particularly for landslides along mountainous highways. Time-series InSAR has been used to detect and track landslide activity along road corridors [21,22] and to support/validate landslide susceptibility mapping and hazard zoning along strategic routes such as the Karakoram Highway [23]. Case-based validation strategies and hybrid frameworks that integrate InSAR-derived deformation evidence into hazard assessment are also emerging [24]. Recent multidisciplinary studies have also demonstrated the value of SBAS-InSAR for characterizing slow-moving slope deformation processes and supporting hazard interpretation in complex geomorphic settings [25]. Additionally, combining SBAS-InSAR with machine learning has been reported to enhance landslide identification and susceptibility mapping along highway corridors [26,27]. Recent studies have also indicated that anthropogenic disturbances associated with infrastructure development and land-surface modification may interact with natural processes, influencing local deformation or instability [28].

Despite these advances, existing studies have mainly focused on regional permafrost deformation mapping, landslide identification, or susceptibility assessment along transportation corridors. Comparatively less attention has been paid to corridor-scale deformation of highway pavement and adjacent roadside areas in frozen-ground environments, particularly along the Dangxiong–Yangbajing section of the G6 Expressway. Therefore, it is necessary to combine long-term deformation monitoring with explanatory-factor analysis to better characterize deformation patterns and their spatial heterogeneity along highway corridors. SBAS-InSAR can provide spatially continuous deformation information, whereas Geodetector can quantify the explanatory power of different factors and their interactions. This integrated framework is therefore suitable for identifying deformation-prone sections and interpreting the associated environmental controls in frozen-ground highway corridors.

In this paper, the Dangxiong–Yangbajing section of the G6 Expressway, located in the frozen-ground environment of the Qinghai–Tibet Plateau, was selected for deformation analysis. This study aims to clarify the spatiotemporal evolution of highway surface deformation and to identify the environmental factors and factor interactions associated with its spatial heterogeneity. Sentinel-1A SBAS-InSAR was used to retrieve multi-year surface deformation from November 2021 to December 2024, and the internal consistency of the deformation results was assessed using statistical metrics. The corridor-scale deformation characteristics of the pavement and adjacent roadside areas were then analyzed in detail. In addition, Geodetector was employed to quantify the explanatory power of terrain, structural, hydrothermal, and ecological factors and to examine their interaction effects. Compared with previous regional-scale studies, the contribution of this work lies in linking multi-year SBAS-InSAR monitoring with Geodetector-based factor and interaction analysis for a highway corridor, thereby providing a remote sensing basis for deformation identification, environmental interpretation, and targeted risk assessment of plateau highways in frozen-ground regions.

## 2. Data and Methods

### 2.1. Study Area

**Figure 1:**
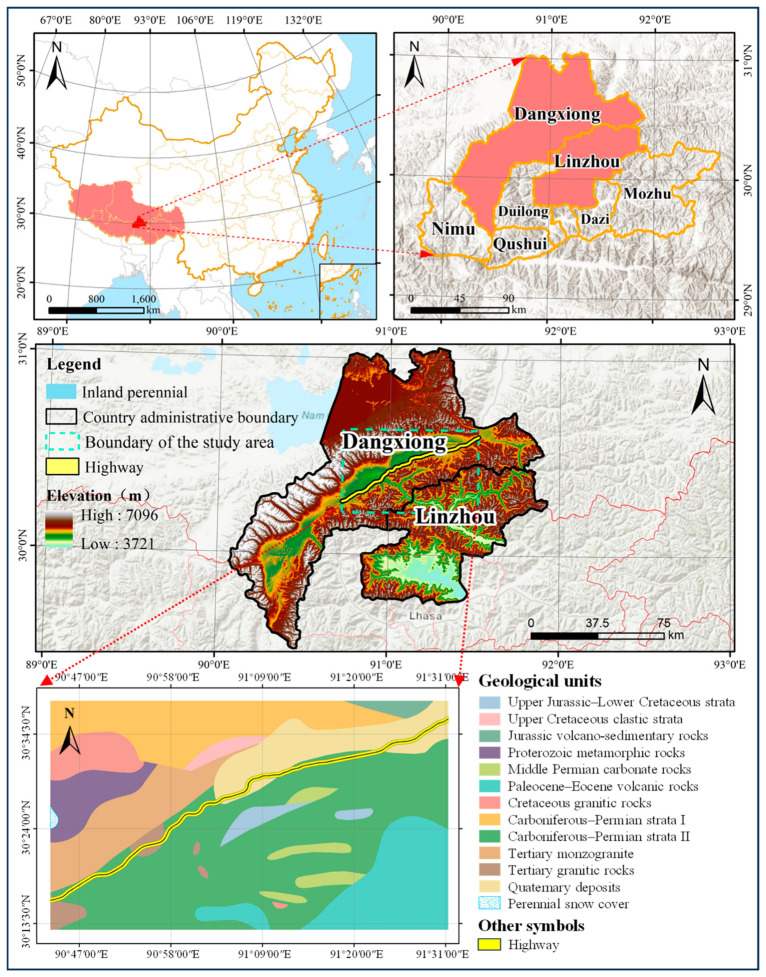
Overview of study area.

The study area is located in the Dangxiong–Yangbajing area of Lhasa City on the Qinghai–Tibet Plateau (center: 91°07′ E, 30°25′ N) and covers approximately 3588 km^2^ (Figure 1). The Dangxiong–Yangbajing section of the G6 Beijing–Lhasa Expressway, a key plateau transport corridor traversing a complex frozen-ground setting, was selected for analysis, with a focus on surface-deformation characteristics along the corridor. Specifically, deformation monitoring and subsequent factor analyses were conducted within a corridor extending 2.0 km on both sides of this ~87 km highway segment. The selected corridor lies within the Dangxiong–Yangbajing Graben, in the central Lhasa Block. The route crosses the Dangxiong and Ningzhong basins, where the topography differs markedly on either side of the graben. The northwestern flank is dominated by the Nyenchen Tanglha Mountains (mean elevation ~6000 m), whereas the southeastern flank is formed by the Pangdo Mountains (mean elevation ~5200 m) [29]. The interior of the graben features relatively flat terrain, constituting the primary route for the highway. The geological and geomorphological setting shown in Figure 1 provides important background for interpreting the spatial heterogeneity of surface deformation.

The region has a high-altitude cold monsoon climate, with cold, dry winters and cool, humid summers; most precipitation occurs in summer. The frozen-ground environment of the Qinghai–Tibet Plateau is characterized by high elevation, strong spatial heterogeneity, and high sensitivity to thermal and hydrological changes. Frozen-ground conditions in the study area include both perennially frozen ground and seasonally frozen ground. Perennially frozen ground is mainly distributed in the high-altitude mountainous areas on both sides of the graben, whereas seasonally frozen ground is predominant along the highway corridor. The active layer undergoes repeated seasonal freezing and thawing, during which ice formation, thawing, water migration, and soil-structure changes may induce frost heave and thaw settlement. These processes can lead to uneven deformation of the roadbed and pose an important engineering-geological risk to the structural stability of this highway segment [30].

### 2.2. Data Sources and Data Description

#### 2.2.1. Radar Data

The SAR dataset comprises 48 C-band single-look complex (SLC) images acquired by the European Space Agency Sentinel-1A satellite. The acquisitions span 24 November 2021 to 26 December 2024. Images were acquired in Interferometric Wide Swath (IW) mode (swath width: 250 km) with vertical transmit and vertical receive (VV) polarization on an ascending orbit and a spatial resolution of 5 m (range) × 20 m (azimuth). During processing, the 30 m Shuttle Radar Topography Mission (SRTM) Digital Elevation Model (DEM) provided by the National Aeronautics and Space Administration was used to remove the topographic phase from the interferograms and to provide consistent elevation information for the regional-scale analysis. Precise Orbit Ephemerides were applied to refine the satellite orbit, improving baseline estimation. Key acquisition and processing parameters are summarized in Table 1.

#### 2.2.2. Image Factor Data

**Figure 2:**
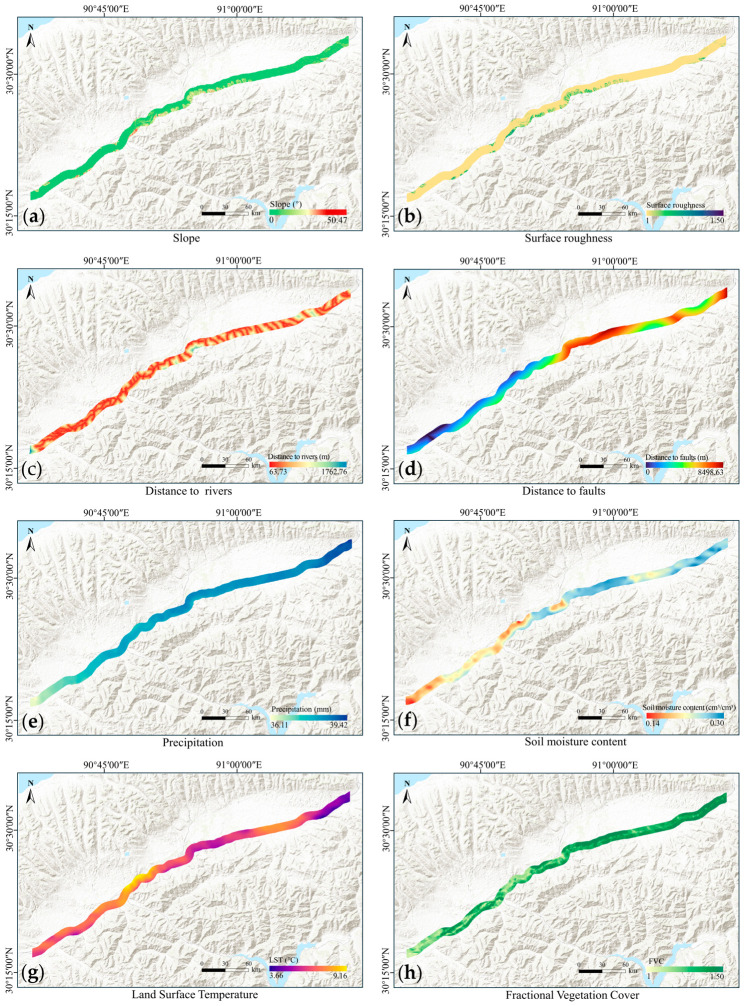
Spatial distribution of explanatory factors: (**a**) Spatial distribution of slope; (**b**) Spatial distribution of surface roughness; (**c**) Spatial distribution of distance to rivers; (**d**) Spatial distribution of distance to faults; (**e**) Spatial distribution of precipitation; (**f**) Spatial distribution of soil moisture; (**g**) Spatial distribution of land surface temperature; (**h**) Spatial distribution of fractional vegetation cover.

To investigate the potential environmental associations with surface deformation in the study area, eight explanatory variables were selected to represent terrain conditions, structural background, hydrothermal factors, and surface ecological characteristics: slope, surface roughness, distance to rivers, distance to faults, surface soil moisture, precipitation, LST, and FVC. The spatial distribution characteristics of each variable are illustrated in Figure 2.

Data sources and preprocessing procedures for the explanatory factors are summarized as follows. Slope and surface roughness were derived from the 30 m SRTM DEM. Distance to rivers and distance to faults were calculated from river and fault vector data, respectively. Precipitation was obtained from the China 1 km Resolution Monthly Precipitation Dataset [31], while surface soil moisture was derived from the China 1 km Resolution Daily All-weather Surface Soil Moisture Dataset [32,33]. Multi-year mean values from 2021 to 2024 were used for precipitation and soil moisture to represent average hydroclimatic conditions during the monitoring period. LST was obtained from the MODIS MOD11A2 product, and FVC was derived from a regional vegetation-cover dataset [34]. All explanatory factor layers were preprocessed to maintain spatial consistency and were resampled to 30 m resolution before Geodetector analysis.

### 2.3. Methods

#### 2.3.1. SBAS-InSAR Technology

SBAS-InSAR effectively suppresses geometric and temporal decorrelation effects caused by long baselines by combining interferometric pairs with shorter spatio-temporal baselines [16]. This capability makes SBAS-InSAR well suited for monitoring slow surface deformation over large areas and long time spans. The fundamental principle of SBAS-InSAR technology is as follows:

The SAR image acquired on 23 June 2023 was selected as the super-master image for constructing the interferometric network. The super-master image was selected according to its central position within the acquisition period and its relatively short temporal and spatial baselines with respect to the other SAR images, which helped improve the overall coherence of the interferometric network. In this study, the maximum temporal baseline and maximum normal baseline were set to 180 days and 5% of the critical baseline, respectively. Goldstein filtering was applied to the differential interferograms before phase unwrapping to suppress phase noise and improve phase continuity. Phase unwrapping was then performed using the minimum-cost-flow method. These processing settings were used to obtain a stable SBAS-InSAR time-series solution for the study area.

Based on these spatio-temporal baseline thresholds, *M* differential interferometric pairs were selected. For an interferogram formed from a master image at *t*_A_ and a slave image at *t*_B_, the unwrapped phase *δϕ_j_*(*x*,*y*) can be written as follows:

(1) $$\delta \phi_{j}\left(x,y\right)=\phi \left(t_{B},x,y\right)-\phi \left(t_{A},x,y\right)\approx \frac{4\pi }{\lambda }\left[d\left(t_{B},x,y\right)-d\left(t_{A},x,y\right)\right]$$

where *λ* is the radar wavelength, and *d*(*t*_B_,*x*,*y*) and *d*(*t*_A_,*x*,*y*) denote the cumulative line-of-sight (LOS) deformation of pixel (*x*,*y*) relative to the initial epoch (*t*_0_), evaluated at acquisition times (*t*_B_) and (*t*_A_), respectively. Because only ascending-orbit Sentinel-1A data were used, the LOS deformation was projected to an approximate vertical component using the radar incidence angle for the subsequent settlement analysis.

Following preprocessing steps such as orbit refinement and topographic phase removal, the phase observations from *M* interferometric pairs can be combined to form the following system of linear equations:

(2) Aϕ=δϕ

where *δϕ* is an *M* × 1 observation vector containing the unwrapped differential interferometric phases. *ϕ* is the *N* × 1 vector of unknown parameters, representing the cumulative deformation phase at *N* acquisition epochs. *A* is an *M* × *N* design matrix whose entries are defined by the indices of the primary and secondary images in each interferometric pair. Each row of *A* contains two nonzero entries (−1 and +1), indicating the temporal positions of the primary and secondary images, respectively.

As the set of baseline-thresholded interferometric pairs may contain independent subsets, matrix *A* is typically rank-deficient, rendering the solution non-unique. A stable minimum-norm least-squares estimate can be obtained using singular value decomposition. After estimating the cumulative deformation *d*(*t_i_*) at each epoch, the mean annual deformation rate *v* over the monitoring period was obtained by linear regression of the deformation time series:

(3) $$v=\frac{\sum_{i=1}^{N}\left(t_{i}-\bar{t}\right)\left(d_{i}-\bar{d}\right)}{\sum_{i=1}^{N}{\left(t_{i}-\bar{t}\right)}^{2}}$$

in which, $\bar{t}$ and $\bar{d}$ denote the mean values of time and cumulative deformation, respectively.

**Figure 3:**
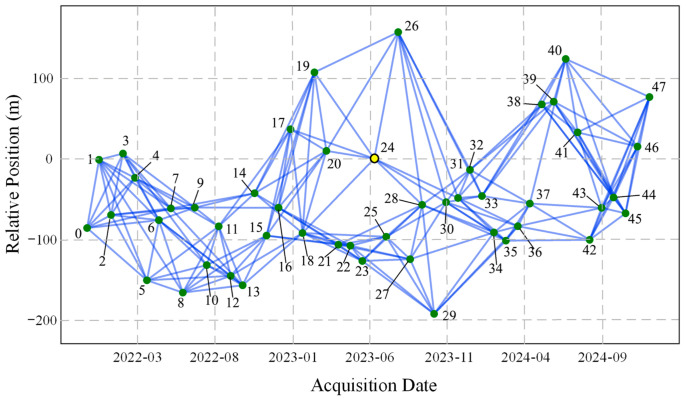
Time-space baseline map.

Based on the above baseline thresholds, the time–space baseline distribution of the SAR acquisitions is shown in Figure 3.

#### 2.3.2. Geodetector

This study employs the Geodetector model to quantify spatially stratified heterogeneity (SSH) in surface deformation and to examine its potential drivers [35]. Unlike traditional regression methods, Geodetector does not assume linear relationships and is less sensitive to multicollinearity among predictors, making it suitable for diagnosing complex geographic processes. Given the requirements for explanatory variable types in the Geodetector model, this study first employed the natural break method (Jenks) to discretize continuous driving factors, dividing them into eight spatial strata to construct a spatially stratified dataset. The eight-class scheme was selected to balance the representation of spatial heterogeneity and the sample size within each stratum. Applying the same number of strata to all explanatory factors also ensured consistency and comparability in the Geodetector analysis. Based on this, a quantitative analysis of the spatial differentiation characteristics of surface deformation was achieved by comparing the variance within each stratum against the overall variance of the study area.

Based on the stratified dataset, the factor detector and interaction detector were applied to assess the effects of individual drivers and their interactions. The factor detector uses the *q* statistic to quantify the extent to which each factor explains the spatial differentiation of surface deformation. The *q* value ranges from 0 to 1; larger values indicate stronger explanatory power for SSH. The *q* value is computed as follows:

(4) $$q=1-\frac{\sum_{h=1}^{L}N_{h}\sigma_{h}^{2}}{N_{\sigma^{2}}}$$

where *q* denotes the explanatory power of factor *X* for the deformation rate *Y* and ranges from 0 to 1. Notably, larger *q* values indicate that factor *X* explains a greater proportion of the spatial stratified heterogeneity of deformation rate *Y*. Here, *h* indexes the strata of factor *X*; *N_h_* is the number of samples (pixels) in stratum *h*, *N* is the total number of samples (pixels), $\sigma_{h}^{2}$ is the variance of deformation rate *Y* within stratum *h*, and *σ*^2^ is the variance of *Y* over the entire study area.

The statistical significance of the *q* statistic was assessed using a permutation test, and factors with *p* < 0.05 were considered statistically significant.

The interaction detector evaluates whether two factors jointly enhance or weaken explanatory power by comparing each single factor *q* with the interaction *q* obtained from their combined stratification. Based on the relative magnitude of the interaction *q*, pairwise relationships are classified into five types according to the Geodetector interaction detector proposed by Wang and Xu [35] (Table 2). This analysis helps identify nonlinear and synergistic effects among factors that contribute to the spatial heterogeneity of deformation rate.

### 2.4. Internal Consistency Assessment of SBAS-InSAR Results

Validation of InSAR-derived deformation is commonly performed by comparison with leveling or GNSS observations [36]. However, the complex terrain of the Tibetan Plateau and limited ground-monitoring conditions make it difficult to obtain high-precision in situ observations that are temporally consistent with the SAR acquisitions. In the absence of external reference data, internal statistical metrics can provide supplementary information on the internal consistency of the InSAR estimates [37,38]. Given the unavailability of leveling and GNSS observation data within the study area, this paper employs the root mean square error (RMSE) of the estimated annual average deformation rate as an internal consistency indicator. This metric summarizes residual variability and internal consistency, supporting the credibility of the deformation estimates.

**Figure 4:**
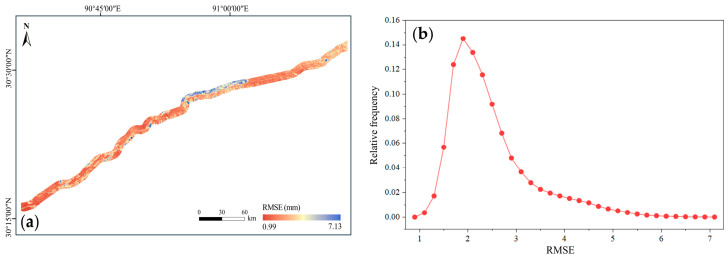
Internal consistency assessment of SBAS-InSAR results: (**a**) RMSE raster map; (**b**) Frequency distribution of RMSE.

Figure 4a shows the spatial distribution of the RMSE of the mean annual deformation rate across the study area. The RMSE is relatively uniform across the region, and most areas show low values. Elevated RMSE values occur only in localized zones, likely due to strong topographic relief, low interferometric coherence, or residual atmospheric delays. Figure 4b shows the frequency distribution of RMSE. The histogram is dominated by low RMSE values, indicating that most pixels have relatively small residual errors. Only a small proportion of pixels fall within higher RMSE intervals, suggesting that larger uncertainties are spatially limited rather than widespread across the study area. The absence of a pronounced long tail or obvious abnormal peak further indicates that no systematic large-error pattern is present in the SBAS-InSAR results. Therefore, the RMSE frequency distribution supports the internal consistency of the retrieved mean annual deformation rates. However, it should be regarded as an internal assessment rather than an independent validation of absolute accuracy.

Taken together, the spatial pattern and frequency distribution of RMSE indicate that the SBAS-InSAR inversion exhibits generally stable internal consistency across the study area, with relatively higher uncertainties confined to limited local zones. On this basis, the retrieved deformation results were used to analyze corridor-scale spatial patterns, temporal evolution, and their associations with environmental factors.

## 3. Results and Analysis

### 3.1. Deformation Identification and Feature Analysis Along the Highway

#### 3.1.1. Identification and Extraction of Typical Deformation Zones

**Figure 5:**
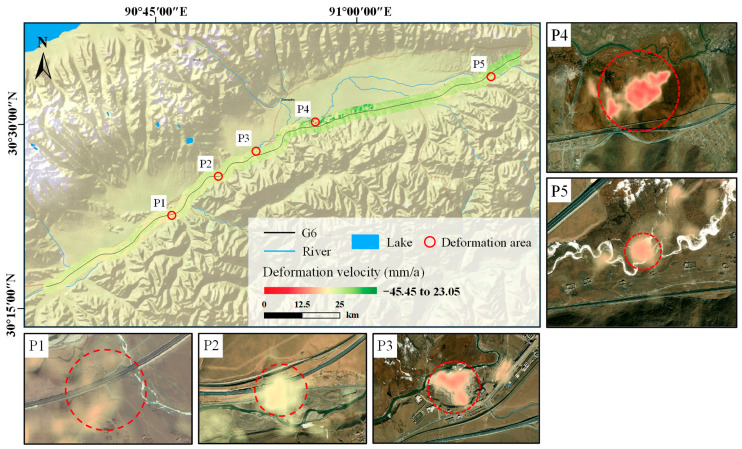
Deformation rate along the Highway.

The vertical annual average deformation rates are shown in Figure 5, where negative values indicate subsidence and positive values indicate uplift. After hotspot analysis was used to screen out error-prone and stable areas [39], optical imagery was incorporated for comparison and verification, and five representative deformation zones (P1–P5; Figure 5) were selected for subsequent analysis. Two of these zones directly traverse or adjoin the highway, constituting the primary surface deformation areas of interest in this research. These deformation zones are distributed in different local geomorphological and hydro-environmental settings, suggesting that their spatial heterogeneity may be associated with variations in terrain position, drainage conditions, and near-surface moisture environments. Among them, P1 and P2 were emphasized in the subsequent analysis because they directly intersect or adjoin the highway corridor.

Across the study area, deformation rates range from approximately −45.45 mm/a to 23.05 mm/a, while most pixels fall between −5.16 mm/a and 4.81 mm/a, indicating broadly stable conditions. Deformation is more pronounced on both sides of the highway than on the roadway itself, with maximum cumulative deformation reaching 161.2 mm. This contrast may be related to differences in surface structure and environmental exposure. Compared with the pavement and embankment body, which are relatively compacted and structurally protected, adjacent natural ground and side slopes are more directly affected by seasonal freeze–thaw processes, rainfall infiltration, runoff convergence, and local moisture accumulation, making them more susceptible to uneven settlement. The deformation near Dangxiong County is the most complex, characterized by a wide range of surface deformation and relatively high deformation rates and magnitudes. In other sections, differential settlement and minor uplift occur on both sides of the highway; however, these deformations do not appear to affect the roadway directly. The largest deformation is observed near Jia Gen Village (P4 in Figure 5), where a pronounced subsidence bowl has developed.

#### 3.1.2. Spatio-Temporal Distribution Characteristics of Pavement Deformation

**Figure 6:**
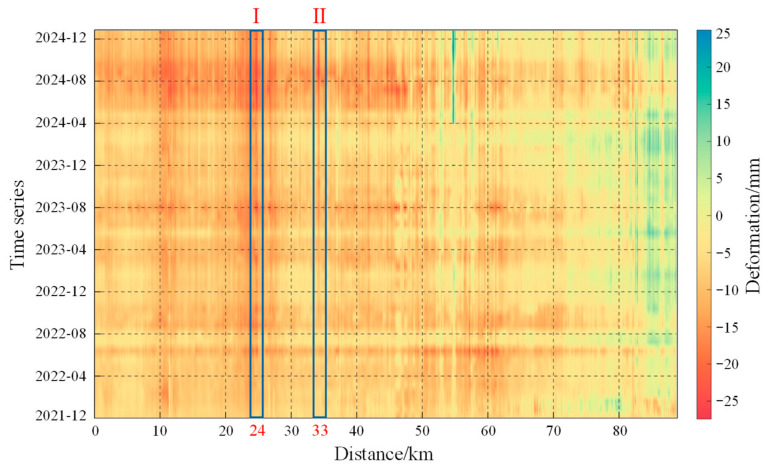
Longitudinal profile deformation of the highway pavement.

To visualize pavement deformation along the highway, a time-series longitudinal deformation profile was constructed from the monitoring results (Figure 6). Deformation values were sampled at 100 m intervals along the route, with the starting point set to 0 km. The results show pronounced variability in deformation with respect to both distance along the route and time.

Two sections with relatively large deformation were identified in the first half of the route (0 km~55 km; Sections I and II in Figure 6). Section I (23 km~25 km) shows the largest pavement deformation along the route, and the representative zone P1 lies within this interval. Deformation in this section is dominated by settlement and increases gradually over time. Settlement reached a maximum in August 2024, followed by a slight rebound, and then stabilized between 0 and −10 mm. Section I was therefore selected for detailed analysis in the following sections. Section II (32 km~35 km) exhibits limited deformation and shows no clear trend of sustained increase. The latter half of the route (60 km~88 km) remains generally stable, with no significant areas of concentrated deformation identified. Fluctuations over the monitoring period are small, and final deformation values remain within −10 mm to 10 mm.

**Figure 7:**
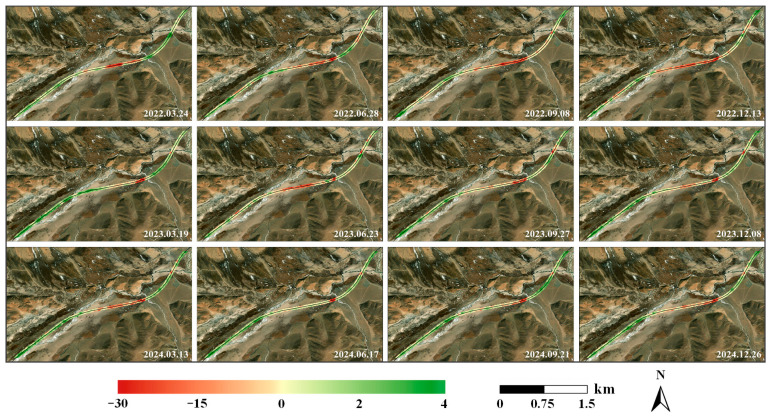
The spatiotemporal variation in cumulative deformation.

To characterize the spatiotemporal evolution of pavement deformation in Section I (Figure 6), we mapped cumulative deformation along this segment for each acquisition epoch relative to the initial reference date (Figure 7). The results show that the most pronounced subsidence occurd in Area P1 near Maling Village along the G6 Expressway. Temporally, subsidence in P1 initiated in March 2022 and subsequently intensified, with both the affected extent and cumulative subsidence increasing progressively. The deformation reached a maximum in June 2024, after which the subsidence rate decreased markedly, and the deformation pattern became largely stable.

## 4. Discussion

The results show that surface deformation along the highway corridor is spatially heterogeneous, with localized settlement occurring in pavement-adjacent and roadside areas. This section discusses the possible factors associated with this deformation pattern, focusing on terrain, structural, hydrothermal, and ecological conditions and their interactions.

### 4.1. Analysis of Potential Drivers of Surface Deformation Along the G6 Expressway

Compared with previous permafrost deformation and plateau highway monitoring studies, the present results further highlight the corridor-scale contrast between pavement and roadside deformation along the G6 Expressway and provide an environmental interpretation of this spatial heterogeneity through Geodetector analysis.

Using the Geodetector factor detector, the explanatory power of environmental factors for the spatial stratified heterogeneity of surface deformation was quantified. Table 3 summarizes the factor-detector results for drivers of subsidence along the highway corridor. Factors differ in their explanatory power for the mean annual deformation rate. In the Geodetector model, the *q* value represents the explanatory power of an explanatory factor for the spatial stratified heterogeneity of the dependent variable. Therefore, a larger *q* value indicates a stronger spatial association between the factor stratification and the deformation pattern [35,40].

Overall, the factor-detector results indicate that the spatial heterogeneity of surface deformation along the highway corridor was associated with multiple environmental factors rather than a single dominant factor. Among the eight explanatory variables, distance to faults exhibited the highest explanatory power, with a *q* value of 0.259, followed by soil moisture, with a *q* value of 0.204. FVC, precipitation, and LST showed moderate explanatory power, with *q* values of 0.130, 0.116, and 0.115, respectively. In contrast, distance to rivers, slope, and surface roughness showed relatively weak single-factor explanatory power, with *q* values lower than 0.03.

The relatively high *q* value of distance to faults indicates that the spatial stratification of this structural factor corresponds well with the spatial heterogeneity of deformation. This suggests that geological background plays an important role in shaping the deformation pattern along the highway corridor. In fault-related areas, differences in rock mass integrity, lithological boundaries, structural weakness, and local hydrogeological conditions can modify the redistribution of water and heat, thereby affecting the mechanical response of near-surface materials under freeze–thaw conditions. Soil moisture also showed relatively high explanatory power, further indicating the importance of near-surface moisture conditions in deformation development. Variations in soil moisture can influence ground-ice formation and melting, pore-water pressure, and the strength of subgrade and slope materials during seasonal freeze–thaw cycles. Although precipitation and LST did not show the highest single-factor *q* values, they remain important hydrothermal variables because they regulate moisture supply and surface thermal conditions. FVC may indirectly influence deformation by modifying evapotranspiration, the surface energy balance, and near-surface moisture retention.

Therefore, the factor-detector results suggest that the deformation pattern is better explained by the combined influence of geological background, soil moisture, vegetation cover, and hydrothermal conditions than by an isolated climatic or topographic factor. Since the *q* statistic reflects the explanatory power of factor stratification for spatial heterogeneity rather than a direct causal contribution, the results should be interpreted as evidence of spatial association. This interpretation provides the basis for the subsequent interaction-detector analysis, which further examines whether paired factors enhance the explanatory power for deformation heterogeneity.

**Figure 8:**
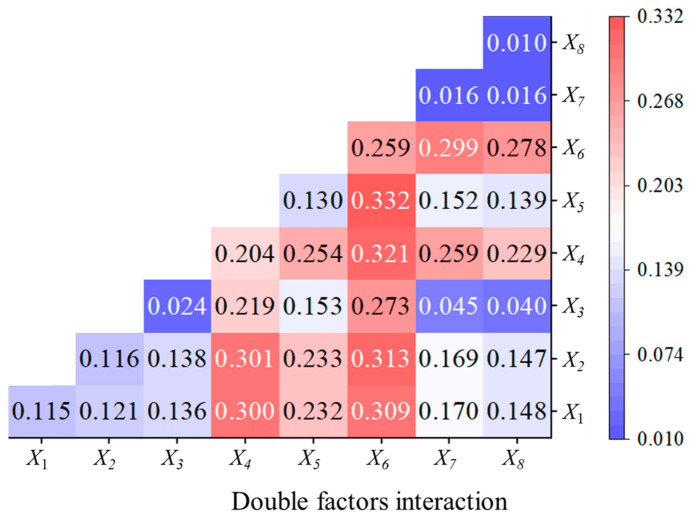
Interaction detection result heatmap.

Building on the single-factor detection, the Geodetector interaction detector was applied to evaluate the combined effects of paired environmental factors on surface deformation. The interaction detector results are summarized in Table 4. To facilitate interpretation, the interaction matrix was further visualized as a heatmap (Figure 8). Overall, the interaction *q* values generally exceeded the corresponding single-factor *q* values, indicating that the explanatory power of deformation heterogeneity was enhanced when two factors acted together. This pattern suggests that surface deformation along the highway corridor was more closely associated with coupled environmental conditions than with any single factor alone.

The highest interaction *q* value was observed for FVC and distance to faults (*q* = 0.332). Other high-value interactions were mainly related to distance to faults, including soil moisture, precipitation, and LST, with *q* values of 0.321, 0.313, and 0.309, respectively. The interactions between soil moisture and precipitation (*q* = 0.301) and between soil moisture and LST (*q* = 0.300) were also relatively strong. These high interaction *q* values indicate that deformation heterogeneity is not controlled by a single environmental factor alone but is more strongly associated with the coupling between fault-related geological background and near-surface environmental conditions. In fault-related zones, structural discontinuities and local hydrogeological conditions may regulate water migration and heat redistribution. Meanwhile, vegetation cover can modify evapotranspiration, surface energy balance, and near-surface moisture retention. This is consistent with the relatively high explanatory power of the interaction between FVC and distance to faults. Meanwhile, the strong interactions involving soil moisture, precipitation, and LST further suggest that hydrothermal processes remain important in shaping deformation patterns. Increased precipitation can enhance infiltration and near-surface moisture accumulation, whereas higher surface temperature can accelerate seasonal thawing and alter the thermal state of frozen or seasonally frozen ground. During freeze–thaw cycles, water migration, ice formation and melting, and the redistribution of pore water can weaken soil structure and reduce the bearing capacity of subgrade and slope materials [30,41]. Therefore, the interaction-detector results further suggest that localized settlement is better explained by the coupling of fault-related geological settings, vegetation-related surface conditions, and hydrothermal variations.

In summary, the spatial pattern of surface deformation reflects the combined effects of multiple interacting environmental factors. Distance to faults and soil moisture showed relatively higher single-factor explanatory power. Meanwhile, interactions among fault-related conditions, FVC, soil moisture, precipitation, and LST further enhanced the explanation of deformation heterogeneity.

### 4.2. Sequential Analysis of Surface Deformation in Typical Regions

The preceding Geodetector analysis suggests that surface deformation is associated with multi-factor coupling rather than being controlled by a single environmental factor. Although precipitation and LST did not show the highest single-factor explanatory power, they remain important hydrothermal variables. They may contribute to deformation evolution through their interactions with soil moisture and fault-related conditions. Because the monitoring period spans more than three years, the deformation time series includes repeated seasonal freeze–thaw cycles. Accordingly, zones P1 and P2 (Figure 5) were selected for time-series analysis. Deformation was examined together with coincident precipitation and LST to discuss the temporal correspondence between settlement evolution and seasonal hydrothermal variations.

**Figure 9:**
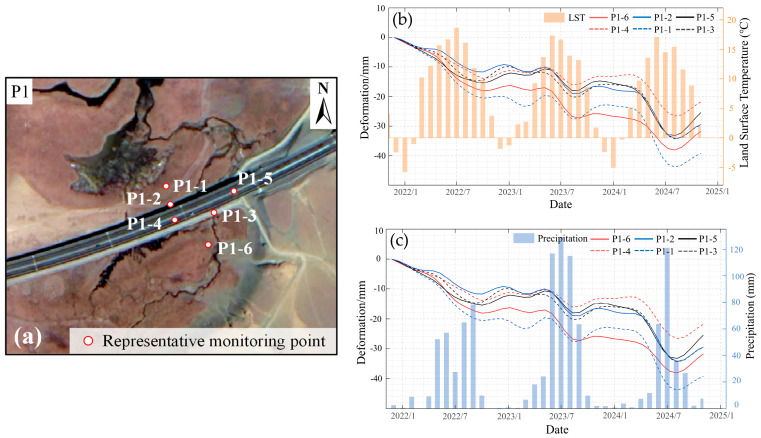
Representative monitoring points and time series analysis of Area P1: (**a**) Distribution of representative monitoring points in Area P1; (**b**) Time series of deformation and LST at the monitoring points (P1-1–P1-6) in Area P1; (**c**) Time series of deformation and precipitation at the monitoring points (P1-1–P1-6) in Area P1.

The representative zone P1, near Ma Ling Village. Six monitoring points were selected for time-series analysis. (Figure 9) P1-1 and P1-6 are on natural ground adjacent to the highway, P1-4 and P1-5 are on the roadway, and P1-2 and P1-3 are on the slopes flanking the highway. The time series shows that subsidence dominated at all points throughout the monitoring period. Deformation curves from different locations display similar temporal patterns, indicating broadly consistent responses between the roadway and adjacent ground over time.

Two phases of accelerated settlement were identified during the monitoring period: 30 May 2023 to 10 August 2023 and 6 April 2024 to 11 July 2024. The first acceleration phase coincided with the peak summer rainfall period. Sustained precipitation increased soil moisture, thereby weakening the mechanical properties of the subgrade and slope-protection materials. Consequently, settlement accelerated under combined self-weight and traffic loading. The second phase lasted longer and spanned multiple freeze–thaw cycles. Its evolution shows a temporal correspondence with seasonal freeze–thaw processes: near-surface soils heave during winter freezing. As temperatures rise, ice melts, and water redistributes, making the soil structure looser and less stable. Snowmelt and spring rainfall infiltration further increase moisture availability, promoting gradual accumulation of thaw settlement. Overall, deformation in Area P1 shows a temporal correspondence with precipitation variability and seasonal surface-temperature fluctuations. This pattern is consistent with the possible influence of rainfall infiltration and freeze–thaw-related hydrothermal changes on cumulative settlement in this region.

**Figure 10:**
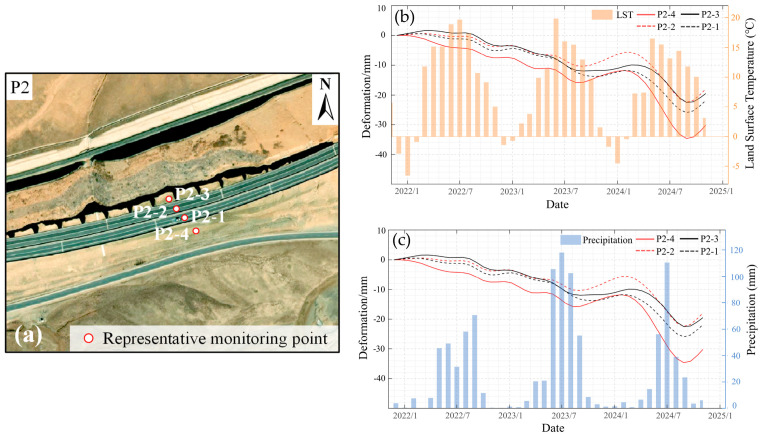
Representative monitoring points and time series analysis of Area P2: (**a**) Distribution of representative monitoring points in Area P2; (**b**) Time series of deformation and LST at the monitoring points (P2-1–P2-4) in Area P2; (**c**) Time series of deformation and precipitation at the monitoring points (P2-1–P2-4) in Area P2.

Zone P2 (Figure 10) is located near the junction of the G6 Expressway and the G109 National Highway, with G109 lying south of G6. Four representative points were selected for time-series analysis: P2-1 and P2-2 on the highway carriageway, and P2-3 and P2-4 on the left and right embankments, respectively. Time-series curves show similar trends across the four points. Settlement was relatively stable from the start of monitoring to August 2023, accelerated between 13 March 2024 and 28 August 2024, and then decelerated and stabilized at a near-constant rate.

The settlement time series shows clear phase-dependent behavior associated with variations in precipitation and LST. Accelerated settlement generally coincides with higher precipitation and sustained increases in surface temperature. Heavy rainfall increases water infiltration into near-surface soils. Meanwhile, rising air and surface temperatures promote thawing and water redistribution, altering soil moisture and structure. During this phase, settlement rates were markedly higher than in earlier periods, showing a temporal correspondence with enhanced precipitation and rising surface temperature. Spatially, the Quhe River lies ~200 m south of the deformation zone, and areas with larger settlements are generally closer to the river. Settlement magnitude increases as distance to the river decreases, indicating a clear spatial gradient. This pattern suggests that river recharge–driven groundwater fluctuations may modulate settlement on the southern side of the road. Coupled rainfall infiltration and seasonal temperature variability may further amplify the settlement response of the southern slope protection.

Overall, the temporal evolution of subsidence deformation in Area P2 appears to be jointly associated with precipitation variability, surface-temperature warming, and proximity to the river, reflecting controls acting across both time and space. Changes in hydrothermal conditions and groundwater processes may have contributed to the sustained subsidence evolution in this region, together with local geomorphic and hydrogeological conditions.

## 5. Conclusions

This study used 48 Sentinel-1A images acquired from an ascending orbit and employed SBAS-InSAR to retrieve surface deformation rates and deformation time series within a 2.0 km buffer on both sides of the Dangxiong–Yangbajing section of the G6 Expressway from 24 November 2021 to 26 December 2024. By integrating SBAS-InSAR deformation monitoring with Geodetector factor and interaction analyses, this study examined the spatiotemporal evolution of highway surface deformation and its environmental associations in a plateau frozen-ground region. The main conclusions are as follows:

(1) Most areas within the study corridor remained relatively stable, with deformation rates mainly concentrated between −5.16 and 4.81 mm/a. However, pronounced subsidence occured in several localized zones, with a maximum rate of approximately −45.45 mm/a.
(2) Deformation along the highway is spatially heterogeneous, exhibiting localized subsidence clusters; moreover, deformation amplitudes on both sides of the highway generally exceed those on the pavement.
(3) Through hotspot analysis and verification using optical imagery, five typical deformation zones (P1–P5) were identified. Among these, P1 and P2—which intersect with or are adjacent to the highway—constitute the primary areas of risk concern.
(4) Geodetector results suggest that subsidence heterogeneity is better explained by multi-factor coupling than by a single dominant factor. Distance to faults and soil moisture showed higher single-factor explanatory power, and their interactions with FVC, precipitation, and LST further enhanced deformation heterogeneity.

Overall, this study provides a remote sensing framework at the corridor scale for identifying highway deformation and interpreting its environmental associations in plateau frozen-ground regions, offering practical support for targeted hazard assessment and maintenance planning of transportation infrastructure.

## Figures and Tables

**Table 1:** Sentinel-1A dataset parameters.

Satellite	Sentinel-1A (SLC)
Acquisition dates	24 November 2021–26 December 2024
Number of scenes	48
Beam	IW
Polarization	VV
Orbit	Ascending
Mean incidence angle(degree)	39.6
Path	41
Frame	95

**Table 2:** Types of interaction between two covariates.

Criterion	Interaction Type
*q*(*X*_1_ ∩ *X*_2_) < min (*q*(*X*_1_), *q*(*X*_2_))	Nonlinear weakening
min (*q*(*X*_1_), *q*(*X*_2_)) < *q*(*X*_1_ ∩ *X*_2_) < max(*q*(*X*_1_), *q*(*X*_2_))	Uni-factor nonlinear weakening
*q*(*X*_1_ ∩ *X*_2_) > max(*q*(*X*_1_), *q*(*X*_2_))	Bi-factor enhancement
*q*(*X*_1_ ∩ *X*_2_) = *q*(*X*_1_) + *q*(*X*_2_)	Independence
*q*(*X*_1_ ∩ *X*_2_) > *q*(*X*_1_) + *q*(*X*_2_)	Nonlinear enhancement

**Table 3:** Results of the factor detector.

Factor	*q*	*p*
LST (*X*_1_)	0.115	≤0.001
Precipitation (*X*_2_)	0.116	≤0.001
Distance to rivers (*X*_3_)	0.024	≤0.001
Soil moisture (*X*_4_)	0.204	≤0.001
FVC (*X*_5_)	0.130	≤0.001
Distance to faults (*X*_6_)	0.259	≤0.001
Slope (*X*_7_)	0.016	≤0.001
Surface roughness (*X*_8_)	0.010	≤0.001

**Table 4:** Results of interaction detection.

**Factor**	*X*_1_	*X*_2_	*X*_3_	*X*_4_	*X*_5_	*X*_6_	*X*_7_	*X*_8_
*X*_1_	0.115							
*X*_2_	0.121	0.116						
*X*_3_	0.136	0.138	0.024					
*X*_4_	0.300	0.301	0.219	0.204				
*X*_5_	0.232	0.233	0.153	0.254	0.130			
*X*_6_	0.309	0.313	0.273	0.321	0.332	0.259		
*X*_7_	0.170	0.169	0.045	0.259	0.152	0.299	0.016	
*X*_8_	0.148	0.147	0.040	0.229	0.139	0.278	0.016	0.010

## Data Availability

Some or all data, models, or code that support the findings of this study are available from the corresponding author upon reasonable request.

## Abbreviations

The following abbreviations are used in this manuscript:

InSAR	Interferometric Synthetic Aperture Radar
SAR	Synthetic Aperture Radar
D-InSAR	Differential Interferometric Synthetic Aperture Radar
SBAS-InSAR	Small Baseline Subset InSAR
PS-InSAR	Permanent Scatterer InSAR
LST	Land Surface Temperature
FVC	Fractional Vegetation Cover
GNSS	Global Navigation Satellite System
IW	Interferometric Wide Swath
VV	Vertical Transmit and Vertical Receive
SLC	Single-look Complex
SRTM	Shuttle Radar Topography Mission
DEM	Digital Elevation Model
SSH	Spatially Stratified Heterogeneity
RMSE	Root Mean Square Error
